# Supplementary material for: Are carbon emissions trading and green financial instruments synergistic? -Comprehensive quantitative research based on content analysis
Source: PLoS One. 2024 Mar 7;19(3):e0298601. doi: 10.1371/journal.pone.0298601 (PMC10919601; doi:10.1371/journal.pone.0298601)
Supplement: S1 Data — (ZIP) [file pone.0298601.s001.zip › Data/地级市Z_F工作报告.zip!/░▓╗╒/│n╓▌╩╨/└┤╫╘╣1⁄2╓┌║┼í╛┬φ┐╦╩2╛▌═°í┐_2015.docx]

各位代表：

　　现在，我代表市人民政府，向大会作工作报告，请予审议，并请市政协委员和其他列席人员提出意见。

**一、2014年工作回顾**

　　2014年是我市历史上十分特殊、极不寻常的一年。宏观经济下行压力持续增大，市内几起重大腐败案件连续产生负面影响，给良好的发展局面带来严峻考验。面对困难和挑战，在省委、省政府和市委的坚强领导下，在市人大、市政协的监督和支持下，我们牢牢把握主题主线，不彷徨、不动摇、不折腾、不懈怠，提振精神，团结一心，迎难而上，共克时艰，经济社会发展实现了稳中有为、稳中有进、稳中提质。

　　（一）全力稳增长，发展势头继续向好。

　　一年来，市政府团结带领全市人民，认真贯彻市委各项部署，瞄准既定目标，以承接转移和创新升级引领新型工业化，以现代农业示范园区引领农业现代化，以美好滁城建设引领新型城镇化，坚定信心谋划发展，奋力开拓推动发展，抢抓机遇加快发展。全年实现地区生产总值1184.8亿元，增长9.4%；规模以上工业增加值564.2亿元，增长12.2%；固定资产投资1248.2亿元，增长16%；财政收入203.6亿元，增长13.8%，其中地方收入完成123.6亿元，跃居全省第三；社会消费品零售总额382.3亿元，增长13.3%；外贸进出口22亿美元，增长18.8%；实际利用外商直接投资9.2亿美元，增长27.2%；城镇、农村居民人均可支配收入分别达到22091元和9171元，同口径增长9.1%、12.1%。总量上，地区生产总值、固定资产投资、财政收入、实际利用外资4项指标位居全省第一方阵（前五名）；增速上，社会消费品零售总额、外贸进出口、实际利用外资3项指标位居全省第一方阵。

　　（二）聚力抓改革，发展活力加速释放。

　　弘扬大包干精神，全面深化改革，完成61项改革任务。农村综合改革上，实施了12项涉农改革，基本完成45个试点镇农村土地承包经营权确权登记颁证，省试点县凤阳和市试点县天长的土地确权登记发证工作扎实推进。定远获批为全国农村改革试验区，并被列为全国农田水利设施产权制度改革和创新运行管护机制试点县。全面完成农村信用社改制任务。行政体制改革上，完成县级工商、质监、食品药品监管体制改革和市各大指挥部平台整合，政府机构改革方案获批。衔接落实省政府取消、下放的审批事项56项；开展了第七轮行政审批流程再造，取消前置中介服务22项。拟定市级权力清单和责任清单；公布涉企收费清单，取消、缓征、降标收费111项，年减轻企业负担8000多万元；建成苏滁产业园“一站式”服务中心并实行“2号章”管理。实施了滁城城管体制改革。经济体制改革上，开展国有集体企业改革扫尾工作。加快通关便利化改革，推行“属地申报、属地验放”模式，安康电子公用型保税仓库获批。落实工商登记制度改革，深化供水价格改革，实施保障房后期维护等首批政府购买服务项目。完善国有资本经营预算制度。全面公开市、县两级政府及部门预决算和“三公”经费信息，健全政府性债务管理制度。社会事业改革上，医改工作全省领先，开展了全省最大规模的医用耗材集中采购，定远率先组建医疗集团。教育信息化建设走在全省前列，天长、南谯、琅琊等地积极开展义务教育集团化改革。实施单独两孩政策，计划生育保持全省先进水平。实现城镇医保省内异地就医结算。明光纳入全省城乡基本公共服务衔接并轨试点。其他各项改革稳步推进。

　　（三）强力扩投入，发展后劲有所增强。

　　深入开展“三帮三提”活动，力促项目快签约、快开工、快建设、快投产、快见效。抓招大引强。新引进市外亿元以上项目346个，其中南方黑芝麻、立讯精密工业、华汇热电联产、中兴能源等10亿元以上项目19个，到位资金700亿元。抓项目前期。大唐电厂历经艰难终获国家能源局前期批复，江巷水库通过水利部审查，滁宁轻轨等10个项目列入全省首批PPP试点项目，盐化产业基地等8个项目纳入省上报国家“十三五”重大项目范围。抓项目开工。新开工项目1520个，其中亿元以上项目260个，同德实业、锦汇新材料、道益科技、锦富电子、派罗特克、昇兴包装二期、中广核风电等一批项目启动建设。抓项目竣工。猎豹汽车、永强汽车、西门子新工厂、中普能源四期、九牧科技、养元饮品二期、宝利嘉纺织二期、东鹏特饮、新盛诺光电、德轮橡胶、晋源铜业、天盛重工等190个亿元以上项目竣工投产。抓项目达产。华塑盐化一期、泉盛化工一期、台玻、蜡笔小新、雅客食品、奥特佳压缩机、万佳联众等亿元以上达产工业项目超过100个。抓要素保障。资金上，广开筹资渠道，创新融资产品，实现社会融资总量500亿元。全市贷款余额1045亿元，居全省第五；新增贷款200亿元，其中域内银行新增贷款143亿元，新增存贷比103.8%。直接融资36亿元，市城投14亿元三期债和首期10亿元中期票据、金禾实业2亿元公司债成功发行，10亿元国开行保险资金直投、5.4亿元交行社保基金落实到位，全柴股份定向增发7亿元获证监会核准。扬子地板、禾益化学、华盛控股在“新三板”挂牌，11户企业在区域性股权交易市场挂牌。用地上，扩增量与盘存量并举，积极向上争取指标，全面开展土地“四清”，争取建设用地指标4.6万亩，供应4.5万亩。用工上，举办各类招聘会500场次，帮招员工8.6万人次。用电上，全社会用电量119.5亿千瓦时，增长11.2%，其中工业用电量87.2亿千瓦时，增长16.4%。

　　（四）大力调结构，发展质量稳步提升。

　　在力求做大总量的同时，着力转方式、提质量、增效益，推动三次产业在更高层次上协调发展。狠抓产业转型升级。工业上，规上企业发展到1291户，比年初净增193户，实现产值2250亿元，增长12%。六大主导产业完成产值1850亿元，增长14%。高新技术企业发展到145家，居全省第六，康佳、天康、蓝德等8家企业进入全省百强；完成高新技术产业产值1000亿元，增长17%，总量居全省第三。战略性新兴产业企业发展到223家，居全省第三；完成产值400亿元，增长13%。24家民营企业入围全省民企百强，居全省第三。农业上，粮食生产实现“九连丰”，总产86.1亿斤。天长再获“全国粮食生产先进单位”。成功举办第九届国际树莓大会。新增定远金山和南谯乌衣2家省级现代农业示范区，全椒获批国家现代农业示范区；新增规模以上农产品加工企业42家，总数达313家，实现产值508亿元，增长15.6%。新增土地流转面积30万亩，总面积达212万亩，流转率达35%；新增家庭农场600家，总数达2100家，居全省第二；新增农民专业合作社753家，总数达4485家。机械化作业率达79.5%，高于全省12个百分点。服务业上，净增限上企业（单位）68户，累计达830户。滁州1912、创达义乌商贸城、99广场等投入运营，长城文化创意园部分启用，苏宁广场、亚太五金城、南京湾商贸物流园、明光广大物流园等项目加快建设。城北商贸物流园和南谯现代物流园获批为省级服务业集聚区。市开发区和秦栏镇被确定为省家电和电子产品出口基地，扬子空调、德力玻璃、全柴动力和长兴玩具成为省出口名牌。引进首个电子商务O2O项目——每天惠智慧便利店，天康医疗成为省级电子商务示范企业。凤阳获“中国最具价值文化（遗产）旅游目的地城市”，全椒入选“2014最美中国”旅游目的地。力促企业转型升级。技改投资完成265亿元，增长15%。开润股份等4家企业列入工信部两化融合试点。新增智慧企业238户。市高新技术创业服务中心获批国家级科技企业孵化器，家电设计与制造特色产业基地通过科技部复核，南大光电高纯电子气体项目入选国家科技重大专项。新组建省级工程技术研究中心3家。全市企业技术中心发展到203家，其中国家级2家，实施技术创新项目300项，完成投资30.6亿元。发明专利申请量、授权量、拥有量分别增长59.9%、85.4%和24.5%，居全省第三、第五、第五位。加快园区转型升级。14个省级以上园区实现财政收入占全市1/3、规上工业增加值占全市一半以上。苏滁产业园一期12平方公里基本实现“九通一平”，已引进项目43个，其中外资项目14个。市开发区启动“三次创业”，荣获“中国开发区30年最具投资价值奖”、“安徽省投资环境十佳开发区”。天长开发区获批省级创新型园区，定远、来安、凤阳开发区扩区规划和定远盐化工基地总规通过省政府审批。

　　（五）着力促统筹，发展载体不断完善。

　　坚持走新型城镇化道路，推进城乡统筹发展，城乡面貌又有新变化。美好滁城品质提升。省政府审议通过了滁城新一轮总体规划。“131”组团实施重点项目375个，其中新开工项目166个，完成投资187亿元；中心城区实施重点项目168个，其中新开工项目56个，完成投资66.5亿元。“十路一渠”基本完工，滁城百姓期盼多年的西涧路即将贯通，滁来全一体化重点工程滁州大道正在分段施工，连接琅琊山、高铁站、乌衣新区的洪武路建设已近尾声，连接政务新区和苏滁园区的龙蟠路跨线桥工程进展顺利，城北干线世纪大道加快改造。琅琊山山体覆绿一期工程、永乐路公园建设等完工，醉翁亭核心景区综合整治初见成效，清流河二期、中央公园、龙池街景观工程开工建设。完成37条小街巷、78条老旧道路改造，凤凰三村、汇龙新村、银花新村、北湖小区等老旧小区改造有序推进。垃圾焚烧发电厂调试运营。延长加密公交线路，新投放公交车70辆、“迷你”巴士20台，滁全、滁来城际公交开通。50个站点、1300辆公共自行车投入使用。依法禁止滁城“三小车”客运，着力推进湖心路、天长路、中都大道等重点路段集中整治，市容市貌进一步改善。来安县城的小街小巷综合治理，全椒县城的老襄河沿岸拆迁改造和儒林文化街区建设成绩显著。美好城镇各展风采。天长、明光、定远、凤阳四个县城以“三治三增三提升”为抓手，坚持老城改造、新区拓展、园区突破同步推进，实施各类重点建设项目近300个，完成投资近200亿元。天长建成红草湖公园南区，获全国文明县城提名；明光市区的规划、建设取得重大突破；定远、凤阳县城打通外环，新区建设有新的进展。秦栏跻身“安徽省宜居小镇”，炉桥、汊河、古河、沙河、临淮关、女山湖等12镇入选“全国重点镇”。农村清洁工程实现乡镇全覆盖。美好乡村加快建设。重点抓好10条示范线和48个中心村建设，全面整治100个沿线沿边村，实施村庄建设项目800多个。定远、凤阳、天长被评为全省2013年度美好乡村建设先进县（市）；全椒黄栗树、来安小李庄等入选农业部美丽乡村创建试点；凤阳小岗、天长长山入选“全国生态文化村”；南谯姑塘等获“安徽省宜居村庄”；天长龙岗、明光梅郢等入选省首批传统村落。基础设施逐步完善。徐明高速建成通车，滁淮高速开工建设，滁马高速、宿扬高速、蚌淮高速连接线加速推进，104国道滁汊段（苏滁大道）改造工程过半，全市高速公路建设规模、在建里程和总投资均居全省前列。淮河入江水道加固工程完工，滁河、池河防洪工程加快实施，淮干蚌浮段行洪区调整工程开工，74座小型水库完成除险加固。全年交通投资106亿元、水利投资16.8亿元、能源建设投资30亿元，其中，水利建设投资连续六年全省第一、市政公用设施投资连续四年全省第二。

　　（六）合力保生态，发展环境持续改善。

　　牢固树立既要银海金山、也要绿水青山的发展理念，一手抓保护，一手抓治理，生态优势进一步彰显。节能减排扎实推进。加强制度建设，强化执法监管，能源消费总量、单位GDP能耗和主要污染物减排指标完成年度目标任务。生态建设有序展开。新增造林25万亩，占省下达任务的124%。成功创建国家园林城市，城区绿化覆盖率近40%。我市获评为2014中国生态文明价值城市，纳入省级生态文明先行示范区。全椒被评为全国水生态文明建设试点县，襄河被评为国家级水利风景区。半塔荣获“全国生态文明先进镇”，新增南谯施集等5个省级生态乡镇、来安桃花等6个省级生态村，杨村、张桥、府城、泊岗等7个乡镇荣膺“安徽省森林城镇”，46个村庄荣膺“安徽省森林村庄”，创建省级森林长廊示范段91公里。环境治理力度加大。建立重污染天气监测预警系统，加大秸秆禁烧力度，淘汰黄标车近8000辆，积极参与南京青奥会和国家公祭日环境质量保障工作，省下达的年度大气污染防控指标如期完成。落实水资源管理“三条红线”制度，启动行政区域计划用水工作。开展“三线三边”环境整治，来安农村生活垃圾“组合收运”模式在全国农村生活垃圾治理工作电视电话会议上交流推广。

　　（七）倾力惠民生，发展成果渐趋共享。

　　始终把保障和改善民生摆在突出位置，完成教育卫生、社会保障等13类民生支出230亿元，占财政总支出的85.7%。民生工程上，新开工保障房1.9万套，建成2万套，居全省前列。改造农村危房8900多户。城镇新增就业9.3万人，城镇登记失业率3.3%，低于省控指标1.2个百分点。城镇职工五项保险参保人数达160万人次；新农合平均参合率100%。以船为家渔民上岸工程进展顺利。老村干、老民师等七类“老字号”群体补助及时发放。全椒被列为全国培育新型职业农民试点县。教育上，完成119所义务教育学校标准化建设，基本完成学前教育三年行动计划，天长、南谯通过义务教育基本均衡县国家级督导验收，来安被命名为省级教育强县。文化卫生体育上，市文化馆新馆开馆，市图书馆分馆建成运营，建成农民文化乐园省级点8个，完成13个公共电子阅览室建设任务，实施明中都城国家大遗址公园建设工程。天长、明光蝉联“全国文化先进市”，南谯获评“全国文化先进区”，凤阳、全椒荣获“中国民间文化艺术之乡”；定远被列为全省唯一的农村公共图书服务体系建设试点县。来安成功创建省级卫生应急示范县，南京儿童医院滁州分院投入使用。全民健身工作“三个一”经验在全省推广。我市在省十三届运动会和省七届少数民族运动会上取得佳绩。琅琊山景区对市民免费开放。人口计生上，克服生育政策调整影响，精确指导、强力推进，人口自然增长率控制在6‰以内。凤阳被评为“全国计划生育优质服务先进县”。信访维稳上，推行阳光信访，开展积案化解，加快数字化治安防控体系和社会管理服务信息化平台建设，全省基层综治维稳信访工作中心建设现场会和网上信访现场会在我市召开。安全生产上，狠抓重点行业领域安全监管和“六打六治”行动，事故起数和死亡人数分别下降15%和1%；食品药品安全形势稳中向好，安全监管能力全面提升。同时，圆满完成第九届村委会换届选举工作；被列为全国未成年人社会保护试点市，全省困难群众“救急难”工作试点市；5人荣登“中国好人榜”；琅琊被授予“全国和谐社区建设示范城区”，成为我市首个国家级示范城区。防震减灾综合考核为全国先进和全省第一。我市获批为省首批创新型试点城市。全面做好第三次全国经济普查登记工作。新建53套多要素自动气象站。老龄、红十字会、慈善和残疾人等事业取得新成绩，工会、共青团、妇联、文联、科协等群团组织作用进一步发挥，工商、质监、商检、海关、编制、供销、物价、外事、侨务、消防、保密、档案、地方志、老干部、工商联、民族宗教、国防动员、关心下一代等工作全面推进。

　　（八）努力转作风，自身建设得到加强。

　　认真开展党的群众路线教育实践活动，坚持“三严三实”，扎实整治“四风”，坚决纠正“为官不为”和“为官乱为”问题，在抓常、抓细、抓长上下功夫，进一步改进作风，不断提升服务水平。认真执行市人大及其常委会的决定、决议，主动接受市人大的法律监督、工作监督和市政协的民主监督，广泛听取各民主党派、工商联和社会各界人士的意见。办理人大议案4件、建议124件和政协提案382件，满意和基本满意率达97%。加强规范性文件审查备案，依法行政水平进一步提升。完善信息公开发布机制，政务服务标准化工作通过省级验收，市长热线、市长信箱办理力度增强。全面落实党风廉政建设责任制，强化行政监察和审计监督，严肃查处违法违纪案件，反腐倡廉工作呈现崭新局面！

　　回顾过去一年，我们倍感欣慰的是，尽管形势十分严峻，但全市上下直面困难，众志成城，同心渡难关，全力稳局面。一年来，我们顶住压力，主动担当，保持良好的政治定力和行动自觉，不断增强干部的工作信心、企业家的投资信心、群众的发展信心，把凝聚人心的过程转化为频增决心的过程；一年来，我们正视挑战，精准施策，紧紧扭住发展这个第一要务，坚定不移抓改革、千方百计促开放、聚精会神搞建设，把应对危机的过程转化为争取先机的过程。通过不懈努力，我们稳住了人心，稳住了队伍，稳住了客商，稳住了经济社会发展的良好势头，为滁州更好更快发展、全面进入全省第一方阵奠定了更加坚实的基础。

　　各位代表，同志们，过去一年的挑战前所未有，过去一年的奋斗充满艰辛，过去一年的成绩来之不易！这些成绩的取得，是省委、省政府和市委坚强领导的结果，是市人大、市政协监督、支持的结果，是全市人民团结拼搏的结果。在此，我代表市人民政府，向全市人民，向市人大代表、政协委员和党代会代表，向各民主党派、工商联、无党派人士，向各人民团体、部省驻滁单位和离退休老同志，向驻滁人民解放军、武警官兵、公安政法干警，向所有关心、支持、参与滁州建设的海内外朋友们，致以崇高的敬意和衷心的感谢!

　　回顾过去的一年，我们也清醒地看到，尽管付出了很大努力，但仍有地区生产总值、规模以上工业增加值、固定资产投资等指标未能实现年初预期目标。滁州发展中还存在不少困难和问题，主要表现在：滁城中心城区集聚功能和辐射能力不强，内外两个同城化尚须努力；政府性债务负担不轻，房地产出现结构性过剩，老城改造与群众期望还有不小差距；县域经济总体偏弱，发展不平衡，少数县市区后劲乏力；改革在一些重点领域和关键环节缺少突破，制约发展的体制机制障碍依然存在，市场主体的活力有待充分释放；群众对融资难、融资贵，看病难、看病贵，上学难、上学贵等问题意见仍较集中，影响稳定的因素还有不少；少数政府工作人员对新形势、新政策学习研究不够，程度不同存在不敢担当、本领恐慌问题，老办法不能用、新办法不会用，个别人对八项规定等遵守、执行不自觉、不认真，甚至顶风违纪，等等。对此，我们将高度重视，下大力气加以解决。

**二、2015年工作安排**

　　2015年是全面深化改革的关键之年，是全面推进“三个发展”的开局之年，也是全面完成“十二五”规划的收官之年，做好今年工作，意义重大。面对经济发展的新常态，我们一定要科学研判形势，坚定发展信心，找准发展路径，进一步增强机遇意识、进取意识、责任意识，敢作敢为、善作善为，以逢山开路、遇水搭桥的精神抓推进，以踏石留印、抓铁有痕的劲头抓落实，力促滁州在新的征程上实现新的辉煌。

　　今年工作的指导思想是：全面贯彻党的十八大、十八届三中、四中全会和习近平总书记系列重要讲话精神，认真落实中央经济工作会议和省委九届十次全会、全省经济工作会议部署，坚持稳中奋进、好中加快的工作总基调，坚持冲刺全省第一方阵不动摇，坚持以提高发展质量和效益为中心，全面推动创新发展、绿色发展、健康发展，努力稳增长、转方式、调结构、促改革、防风险、惠民生，确保经济又好又快发展，社会大局和谐稳定。

　　经济社会发展主要预期目标是：地区生产总值增长9.5%左右，固定资产投资增长16%，财政收入增长10%以上，社会消费品零售总额增长13%，外贸进出口增长8.5%，城镇和农村居民人均可支配收入分别增长9.5%和12%，居民消费价格涨幅控制在3.5%左右，城镇登记失业率控制在4.4%以内，人口自然增长率控制在6.5‰以内，完成节能减排任务。

　　新常态有新机遇，新常态有新要求，新常态应有新作为。这些指标的安排，适应了国内外形势的新变化，结合了滁州发展的实际，兼顾了尽力而为与量力而行的原则，既充分考虑了全面建成小康社会的刚性指标要求，也为深化改革、转型升级和改善民生留出了较宽松的空间，有利于引导各方把工作重心聚焦到稳中奋进、好中加快的工作总基调上来。

　　今年，将着力抓好以下几方面工作：

　　（一）发展为要，进一步壮大实体经济。以加快转变发展方式、促进转型升级为主线，突出工业主导，强化农业基础，推进服务业壮大，促进三次产业融合发展。

　　加快新型工业化进程。按照“链形延伸、骨干带动、科技长入、工农融合”的思路，加快推进工业转型升级。一是加长加粗产业链条。实施好传统产业改造升级计划，加快集群化、信息化、品牌化步伐，推动传统块状经济向现代产业集群转型。狠抓产业链招商，建链、补链、强链，推动优势特色产业壮大体量、两头延伸，提升整体竞争力。力争全年六大主导产业实现产值2100亿元，增长12%以上。二是做大做强骨干企业。重点支持规模领先、行业领军、发展势头好的工业企业，推进改造重组、裂变发展，加快打造一批百亿元、50亿元、30亿元企业。力争康佳率先过百亿，博西华、天大、天康早日迈进“百亿俱乐部”，蓝德、全柴、华塑、中普能源等尽快突破50亿元；新增规上企业100户以上，实现工业增加值630亿元以上。进一步加大对小微企业的支持，通过分类指导、梯次培育，千方百计扶大育强促优。引导督促企业不断技改，全年实施重点技改项目300个以上，完成投资300亿元以上。三是依托依靠科技创新。亿元以上工业企业均要建立与科研院所、高等院校以及技术团队的紧密型、常态型、专业型合作，建立企业技术中心、研发中心，实施好人才计划，切实加大对国家级实验室、院士工作站、博士后工作站和各类技术研发机构等扶持力度。支持智能家电、新型材料、新能源汽车等新兴产业发展，加快南大光电、天大铜业、天光半导体、鑫国金属功能材料等项目建设。力争全年战略性新兴产业企业新增20家以上。四是联结联合一二产业。充分认识和发扬滁州工业发展的比较优势在农业这一特长，大力推进工业与农业对接、车间与田间联姻，把农田作为工业第一车间，围绕转化农产品资源，加快培育一批产业化龙头企业，把“农”字头的工业品做精、做特、做大，反过来带动第一车间种养水平的提升。全年各级龙头企业发展到840家，其中规模以上农产品加工企业350家，实现产值570亿元以上。

　　推动现代服务业发展。重点突破电子商务，全力发展文化创意，高度重视房地产业，加快推进服务业发展提速、比重提高、层次提升。电子商务上，坚持政府推动、市场运作、企业主体、典型示范，着力培育2个以上具有较强影响力、完全实现网上交易的电子商务平台。加大政策支持力度，设立1000万元专项资金，扶持和引导电子商务产业发展。大力实施电子商务示范工程，开工建设康佳电子商务产业园等项目，积极创建国家和省级电子商务示范市、示范基地（园区）和示范企业。文化创意上，积极谋划生成一批创意产业项目，依托科教园区、乌衣新区等，探索建立功能定位合理、具有明显特色的创意产业园区或基地。鼓励利用闲置厂房、仓储用房等兴办文创产业，利用工业遗产、建筑遗存发展文创项目。把长城文化创意园作为一号工程，加快推进三期项目，科学谋划后续项目，力争将其打造成为新的城市名片和国内外有广泛影响的东方梦世界。深度挖掘明文化、孝文化、醉翁文化、儒林文化、花鼓文化等地域文化内涵，加快推动醉翁亭景区整治、儒林外史文化城等项目建设。房地产业上，制定针对性更强的政策措施，增强消费动力，规范市场秩序，提升开发质量，推动房地产业持续健康发展。全年完成房产开发投资270亿元。物流业上，加快南京湾商贸物流园、城北商贸物流园等物流聚集区以及全峰快递、鸿耀物流等重点项目建设，打造粤赣皖苏物流节点城市。旅游业上，潜心打造精品旅游线路和目的地景点，丰富旅游业态，重点推进琅琊山、小岗村争创5A级景区，凤阳韭山洞和明皇陵、天长红草湖、来安景华生态园等争创4A级景区，加快推进全椒西环线乡村旅游带、明光女山和老嘉山，以及来安舞彩、南谯皇甫山、琅琊舜田等生态旅游项目建设。养老服务业上，围绕建造老年人宜居家园，积极引导社会资本兴办养老机构，在引进中高端养老养生项目上实现突破，吸引外来人口到滁养老；加强养老基础设施建设，务实做好福利性、公益性养老服务，满足老年人多样化需求。年内建成50个居家养老服务中心（站），改扩建敬老院8个、新增床位600张。

　　提升农业现代化水平。在稳定粮食生产的同时，坚持用工业的理念发展农业，用工业园区的理念打造现代农业示范园区。研究制定相关配套政策，吸引工商企业、金融机构投资农业园区，鼓励农民以土地或劳动力入股。整合涉农项目和资金，主要用于园区建设。加快建设天长永丰、全椒襄河等6个省级农业示范园，打造全省一流精品示范园区；争取定远金山创建国家农业科技示范园区及明光、琅琊创建省级示范园区取得成功。加快推进与中农大、西北农林大、南农大、南林大、安农大等院校合作，提升现代农业发展水平。依托园区开展农业项目招商，引导企业入园集聚发展，新增农业示范园区21家。培育新型经营主体，创新农业经营模式，发展适度规模经营，全年新增家庭农场400家、农民专业合作社600家，新增土地流转面积30万亩以上。

　　振兴县域经济。县域经济是富民强市的基础，也是我市发展“短板”，县域强则全市强。按照重激励、硬约束、严考核原则，力促县域经济加快提升。一是争先进位要有新成效。既要纵向比进步，更要横向比进位，力促各县市区在全省考核中增速争先进、总量进位次、人均上台阶。二是产业培育要有新进展。把培育打造支柱产业、扶植壮大骨干企业作为振兴县域经济的核心，大抓项目，抓大项目，强化发展支撑，积蓄发展后劲。三是城乡面貌要有新变化。坚持因地制宜，通过完善功能、提升品位、突出特色、加强管理等手段，使城乡面貌年年有变化、三年上台阶、五年大变样。四是民生福祉要有新改善。城乡居民收入不断增加，生产生活条件不断改善，群众享受到更多公共服务和更多实惠，幸福指数明显提升。

　　（二）项目为纲，进一步扩大有效投入。恪守经济工作项目化、项目工作责任化的原则，始终把项目摆在经济工作的首要位置，抓在手上，放在心上，落实在行动上。

　　深化开放合作。力争引进市外亿元以上项目400个以上，到位资金800亿元左右。一是研究新对策。适应国家政策调整，顺时应势，对原有政策进行清理，迅速出台新形势下招商新政策、新举措，尽快解决新办法能用、会用、管用的问题，再造招商新优势。二是拓展工作面。坚持全员招商与专业招商并行，发挥好9个专业招商分局和44个县干招商小组作用，推进驻点招商、网上招商、以商招商、产业链招商和园区共建等，一二三产业并举，引资引智引技结合，全力在产业链关键环节和核心项目、外资项目的招引上实现新突破。三是增强软实力。秉承亲商、重商、安商、扶商、富商的理念，全力打造效率更高、服务更优、诚信更佳的投资环境，切实解决企业建设和发展中遇到的困难和问题。力促焕发生物、德威新材料、胜华波汽配、强强实业二期、德力高档玻璃器皿等一批在建项目早竣工、早投产，全年竣工亿元以上工业项目超100个；推动椰牛饮品、德青源蛋品、施耐德变压器、天长缸盖、立光电子等一批重点项目如期开工，全年新开工亿元以上项目300个以上。四是提升融入度。深化区域合作，加强对国家“一带一路”战略研究，积极参与长三角区域分工合作和长江经济带建设，推进与南京同城化、与合肥一体化。紧紧抓住并主动融入南京江北新区，谋划、争取大江北协同发展区成为国家层面的发展战略。

　　加快项目推进。“十二五”即将结束，在抓好在建、在手项目，特别是滁州汽车城、健康食品城、家电制造基地、滁宁轻轨、大唐电厂、江巷水库等十大工程的同时，加强“十三五”重大课题研究，在补短板、增后劲、促均衡、上水平上下功夫，开发储备一批对经济社会发展有支撑引领作用的重大项目，确保全市项目库动态储备亿元以上项目500个以上。特别是围绕大江北新区、农村综合改革试验区、乌衣大学城、长城文创基地等重大平台，信息、生物、环保、智能家电等重大产业，宁西高铁、北沿江铁路、宁合城际轨道、明合高速、引江补淮、窑河复航、山许水库和各类港口等重大基础设施，进行深度谋划，促成更多项目进入国家和省规划范围。

　　提升园区平台。按照产城一体的理念，把各开发园区打造成企业集聚集群、资源优化配置的发展高地。苏滁产业园要继续加快基础设施建设，全力推进产业项目的引入、建设，确保全年新签约、新开工、新投产亿元以上工业项目分别达到20个、15个、10个以上，力争在旗舰型项目的招商上实现零的突破。市开发区要加快“三次创业”步伐，争取规上工业增加值、固定资产投资、财政收入分别增长20%以上，继续领跑全市。推进天长开发区申报国家级开发区，全椒、来安汊河开发区争创省级创新型园区，凤阳•宁国现代产业园建设提速，加快明光、全椒开发区扩容升级，推动定远•肥东合作共建定远盐化工业园、明光•肥西合作共建明光工业园区，并纳入皖北合作共建园区政策范围。

　　破解要素制约。再造用地保障优势，对上争取与对内挖潜并重，使用增量与盘活存量并重，提升土地集约节约利用水平。再造资金保障优势，坚持直接融资与间接融资并举，创新融资方式，力争全年社会融资总量超500亿元；深化政银企合作，争抢信贷规模，调优信贷结构，力争年内新增贷款150亿元；鼓励有条件的企业扩大直接融资，腾出信贷规模；支持已上市公司进行债权融资、股权融资及并购重组，推进浩淼安防、秦栏小贷、龙利得包装等“新三板”挂牌，力争全年上市和“新三板”后备企业达100户以上；大力引进银行、证券、基金、保险等金融机构，完善和发展担保、小贷、典当等机构，探索设立民间融资服务中心，大力发展普惠金融，提高金融服务实体经济和小微企业水平。再造人才保障优势，出台富有竞争力的人才政策，重点引进高层次领军人才和高技能实用人才；实行市县乡村联动，外引、内招、定培并举，出台鼓励外来务工人员购房居住政策，吸引市外劳动力到滁务工定居，让更多转移人口进得来、留得住、过得好。

　　（三）改革为先，进一步提升创新水平。解放思想，着力推动大众创业、万众创新，不断增强治理能力、激发内在动力、释放社会活力，不断增强区域综合实力和核心竞争力。

　　深化重点领域改革。尊重群众首创精神，强化勇于担当意识，找准切入点和突破口，在全面深化改革上取得新进展。加快推进农村综合改革。以农村土地确权为抓手，扎实推进12项涉农改革，完成农村土地确权登记颁证和8个村集体产权制度改革试点任务，推进农村集体“三资”管理网络平台建设，健全县乡村三级土地流转服务体系，扩大农村公共设施“自选、自建、自管、自用”试点范围，推进国有林场改革，争当农村改革排头兵。深入推进行政体制改革。以简政放权为重点，实施好权力清单、责任清单和涉企收费清单制度，探索建立负面清单制度，建设“一库四平台”。进一步取消和下放行政审批事项，扩大政府购买服务范围。深化投资体制改革，大力推行PPP模式，引进社会资本参与城市基础设施建设。深化公共资源交易管理改革。开展公务用车制度改革。着力推进财税金融改革。完善预算制度，加大预算统筹力度。实行政府性债务分类纳入预算管理，切实防范债务风险。调整财政资金支持方式，由过去直接奖补具体项目改为设立专项基金（资金），用于支持产业培育、科技创新、生态环保、土地节约、品牌扶持、人才工程、社会保障等。推进营改增扩围试点。加快金融改革，强化信用体系建设。积极推进社会领域改革。加快户籍管理制度改革，实行居住证制度，鼓励进城务工人员等在城镇落户；加强农民工职业培训、社会保障、权益保护，进一步解决农业转移人口就业、住房、就医、就学、社保等问题，促进基本公共服务均等共享。深化文化、卫生、教育、养老等项改革，稳步推进其他改革。

　　广泛开展大众创业。更新创业理念，培育创业精神，增强创业能力，浓厚创业氛围。把握生产小型化、智能化、专业化等产业组织新特征，进一步降低门槛、加强政策支持，落实“三证合一”，培育壮大各类市场主体。倡导大中专毕业生开展“创业型就业”，鼓励城镇失业人员自主创业，引导农村富余劳动力和退役军人进城、进企务工创业。鼓励外来人员创业，融入滁州，成为新滁州人。引导在外人士返乡创业，实现“人回乡、企回迁、钱回流”。鼓励农民结合美好乡村建设，发展高效农业、品牌农业、生态农业、休闲农业，创办“农家乐”，就地创业。力争全年新增个体工商户2万户、私营企业4000家以上。

　　不断强化创新驱动。加快建立以企业为主体、市场为导向、产学研相结合的技术创新体系。大力发展高新技术产业，力争新认定高新技术企业40家，创新型企业30家，高新技术产业实现产值1100亿元。推动信息化和工业化深度融合，新认定省级以上“两化”融合示范企业6家，新增智慧企业200户以上。开展“发明专利申请攻坚年”活动，新申请发明专利3300件，授权发明专利200件。争取与高校、院所新增合作项目不少于100项。继续扶持中国家用电器研究院、汽车与家电技术及装备研究院、中国质量认证中心华东分中心、国家电子元器件质检中心、石英砂质量检验中心等平台建设，推动轨道交通装备检测中心和盐化工研究院、食品研究院等新型研发实体建设，市科技孵化器二期投入使用。

　　（四）统筹为重，进一步加快城乡建设。推进以人为核心的新型城镇化，让城市和乡村各显特色、各具风采、和谐共生。

　　始终坚持规划引领。坚持规划“一套图”和空间“全覆盖”，依据新版滁城总体规划，加快完成各专项规划及重点地块控制性详规编制工作，强化多规融合，进一步完善规划体系。加强风貌特色规划研究和城市设计引导，塑造特色城市。推进县城和集镇规划全面提升，完善城镇总体规划、控制性详规、专项规划及重点地段设计，彰显城镇魅力。统筹乡村规划，体现田园、生态、宜居特色，建设让人们留恋不舍、记得住乡愁的幸福家园。

　　下力气提升滁城建设水平。突出功能配套，提升城市品质，增强中心城市能级，擦亮“中国亭城”品牌。持续推进“131”组团的内部同城化和与南京的外部同城化，增强城市聚合度。全年实施重点城建项目135个，完成投资85亿元。年内确保苏滁大道、西涧路、洪武路、世纪大道全线贯通，推进滁州大道、龙蟠大道东段等工程。继续完善新城区功能，加快生活配套设施建设。统筹老旧片区、街区、小区改造，各区老旧小区改造2个以上。加快城北汽车站建设，完善城市功能。聚力推进“一山、一河、一城、一湖”四大工程，琅琊山以创建国家5A级景区为引领，加快醉翁亭、丰乐亭景区综合整治，提升深秀湖、琅琊寺等景观，推进龙池街改造，提高名山名气；清流河力推治污、蓄水、绿化等综合整治，逐段布置园林景观，完成园路、广场等配套项目，实现路通、灯亮、水清、景美，提升滁城灵气；滁城古城推行低影响开发模式，完善南湖景观配套，启动城中河拆迁改造工程，展示古城风貌，延续精脉，留住记忆，以古色古香浓郁滁城文气；明湖在完成立项及争取进入国家“十三五”专项规划、省“861”项目笼子的基础上，深化细化区域控规和城市设计，启动水利工程、湿地工程建设，拓展滁城生态空间，涵养城市元气。

　　同步推进县镇村建设。天长、明光、凤阳、定远四个县城要主攻重点项目，完成投资超200亿元。天长要实施好南市区二期6平方公里片区组团开发，加强老城区风貌研究和保护，继续搞好环城河治理。明光要加快城东新区建设，适时向北发展，并注重分块改造老城区。凤阳要坚持新城区完善与老城区改造并举，加大城市主出入口改造建设力度，结合明中都城国家大遗址公园建设，争创国家旅游目的地和省园林城市。定远要启动东部新城建设，推进老城配套、升级，搞好内城河生态环境治理。集镇建设要力戒“夹皮沟”、骑路集，杜绝私人建房，镇当城建，走“块状布局、集中开发、市政配套、功能完善”之路。加快炉桥、汊河、乌衣、铜城、秦栏、临淮关、大墅等重点市镇建设。全面完成去年48个中心村建设任务，组织开展今年45个中心村规划建设，推动“物的新农村”和“人的新农村”建设齐头并进。

　　切实加强城乡管理。进一步理顺、完善滁城管理体制机制，出台滁州城区城市管理考核办法，实现城市管理在时间、空间上全覆盖。牢固树立“八化”理念，推进属地化、网格化、市场化、机械化、数字化、精细化、人性化、长效化管理。围绕“三治三增三提升”，加大城镇环境综合整治力度，着力解决交通不畅、垃圾死角、占道经营、乱搭乱拉等突出问题，严格落实“门前三包”等有效措施。坚决查处违法用地和违章建筑，严格控制新的违建产生。切实加强物业管理，开展老旧小区物业管理规范化行动。强力推进“三线三边”整治，完成300个村垃圾治理，让干净整洁有序的环境常态化。

　　不断完善基础设施。滁马高速、宿扬高速和蚌淮高速连接线建成通车，滁淮高速、滁州港、凤阳港、104国道滁城西段和明光段改建等加快建设，完成交通投资100亿元以上。力争章广风电一期项目建成发电，推进500KV釜山变、220KV韭山变、110KV凤凰变等项目建设，完成能源建设投资40亿元以上。加快推进光纤入户和4G网络建设，完成通信投资8亿元以上。

　　（五）生态为基，进一步推进绿色发展。把生态环境作为滁州决胜未来的核心竞争力，作为市民安居乐业的首要保障。坚持源头严防、过程严管、后果严惩，治标治本多管齐下，让滁州天更蓝、地更绿、山更秀、水更净。

　　打造绿色滁州。开展绿满滁州建设行动。重点围绕城市出入口、绿色长廊、石质荒山等，实施绿化项目80个，新增绿地230万平方米。把实施森林增长工程与发展绿色产业、建设美好乡村相结合，建设一批苗木、花卉、果林、茶药生产基地。完成新增造林20万亩，争创省级森林城市4个、森林城镇7个、森林村庄35个。加大绿色建设推广力度。开展建筑产业现代化综合试点，探索采用标准化设计、工业化生产、装配式施工和信息化管理等方式建造、使用和管理建筑。大力推广绿色建筑、新型墙材、绿色施工和可再生能源应用。加快发展绿色交通，推进公共交通建设，完善城市“绿道”系统和中心城区公共自行车系统。

　　加强生态保护。弘扬生态文化，提高环境保护的公众参与度。深入开展生态镇、生态村等创建活动，新增省级生态县1个、生态乡镇2个以上、生态村3个以上。开展生态修复，启动34处、推进51处矿山治理，完成龙池街宕口、凤阳馒头山、明光明西等矿山治理项目。坚守耕地保护红线，落实耕地保护机制，完成71万亩高标准基本农田建设任务。强化政策导向和利益导向，加强生态补偿等方面的政策创新。

　　强化环境综合治理。实施“四水”共治。治污水，加快污水处理设施建设，开展白塔河、池河、来河、襄河、清流河等河流专项整治；防洪水，推进滁河综合治理，建设滁城城南新区防洪工程，启动沙河集水库除险加固工程，完成2座中型水库、92座小型水库除险加固；保供水，加强重点饮用水源地保护，大力整治工业污染和农业面源污染，确保水质安全；抓节水，鼓励工业企业、绿化灌溉和建筑工地使用中水，进一步提高水资源循环使用率。加大空气污染治理力度，重点治理工业废气、机动车尾气和城市扬尘，切实做好秸秆禁烧和综合利用工作。强化环境监管，对重金属、化学品生产和危险固废处理等重点企业，实行全过程监管和风险防控，及时消除环境安全隐患。

　　（六）民生为本，进一步增进群众福祉。守住底线，突出重点，更加注重保障基本民生，更加注重发展社会事业，更加注重维护和谐稳定。

　　不断提升社会保障水平。全面完成民生工程年度任务。新开工保障房1.95万套，完成8500户农村危房改造。落实积极就业政策，重点做好高校毕业生、城镇就业困难人员就业工作，全年城镇新增就业4.2万人。继续推进全民参保，扩大社会保险覆盖面。适时调整城镇基本医疗保险政策，完善教育、五保、低保等社会救助体系。完成以船为家渔民上岸工程。关爱残疾人，完成贫困白内障患者免费复明手术1200例。切实做好老村干、老民师等七类“老字号”群体生活补助发放工作。

　　切实加快社会事业发展。一是推动教育优先发展。优化教育布局，加大教育投入，开展多种形式的合作办学，全面实施第二轮学前教育三年行动计划，新建公办幼儿园19所、改扩建8所，完成55所义务教育学校标准化建设任务，力争来安、全椒、琅琊通过义务教育基本均衡县国家级督导验收。认真实施特殊教育提升计划。加快职教大市建设，推进城市职业学院、机电工程学校新校区建设，力争天长、明光职教中心和来安职高等通过国家改革发展示范校验收。支持安徽科技学院、滁州学院发展，力争职业技术学院通过省示范性高职院校验收。二是提升医疗卫生服务水平。巩固基层医改成果，探索县乡村医疗卫生机构一体化发展。加强与市外优质医疗资源的合作共建，加大名院、名科、名医引进力度。启用二院新院区。强化城乡医疗卫生基础设施建设，开展基层医疗卫生机构示范化创建活动，加强重大传染病防控。三是扩大文化科技等惠民覆盖面。精心办好第五届中国农民歌会和第五届皖台科技论坛。推进传统媒体和新兴媒体融合发展，弘扬主旋律，传播正能量。加快市博物馆布展，力争市科技馆开馆，统筹推进乡镇综合文化服务中心和村（社区）文化乐园建设。广泛开展全民健身活动，大力推进体育场馆建设。加强人口和计划生育工作，促进人口均衡发展。扎实做好1%人口抽样调查工作，启动我市第三次全国农业普查。持续推进双拥创建工作，力争创建全国双拥模范城“五连冠”获得成功。扩建市殡仪馆并选址建设新陵园。全面推进气象现代化建设，启动皖东强对流监测预警中心工程。认真抓好外事、侨务、对台、宗教、统计、物价、编制、档案、保密、人防、地震、科协、老干部、地方志、红十字会等工作。

　　全力维护和谐稳定。一是抓好信访维稳。顺应信访改革新要求，进一步完善五级群众工作体系，加强人民调解、行政调解和司法调解，推进诉访分离，更加注重运用法治方式化解矛盾、维护稳定。二是加强安全管理。坚持问题导向，强化红线意识和底线思维，建立安全隐患排查治理体系和安全预防控制体系，严格落实安全生产责任制。完善消防安全网格化管理，建立常态化火灾隐患排查机制。强化食品药品安全监管，铁腕整治违法行为，确保群众舌尖上的安全。健全防灾减灾应急体制机制，切实提高事前预警、事中救援、事后恢复的综合处理能力。建立健全舆情引导、监管和应对机制，加强信息和网络安全。三是改进社会治理。坚持系统治理，加强基层基础建设，推进社区管理服务创新。深化平安滁州建设，健全立体化、数字化社会治安防控体系，打击违法犯罪，不断提高群众安全感。

　　着力办好十件实事。遵照市委五届九次全会要求，今后每年市政府向市民承诺，为市民办好十件实事。今年的十件实事包括：开工建设市图书馆；加快市第一人民医院建设（新建急诊内科大楼，南京鼓楼医院滁州分院挂牌，苏滁分院开工建设）；实施滁城内城河改造一期工程；完善滁城城南新区生活配套设施（包括百货超市、幼儿园、菜市场等）；完成滁城棚户区1450户和4个老旧小区改造任务；实施滁城幼儿园布局调整及危房改造工程；开工市福利中心改扩建项目；建设市残疾人服务中心；加快市老年大学及老年人活动场所建设；新建、改造滁城街头游园5个以上和公厕10个以上。

**三、努力建设法治政府**

　　党的十八届四中全会对全面推进依法治国作出了战略部署，开启了建设法治国家的崭新征程。政府系统及其工作人员要忠实履行宪法和法律赋予的神圣职责，加快建设职能科学、权责法定、执法严明、公开公正、廉洁高效、守法诚信的法治政府。

　　树立法治思维。时刻牢记法律红线不可逾越、法律底线不可触碰，自觉将法治理念贯穿到经济社会发展各环节、各领域，切实维护宪法法律权威，全面推进依法行政，运用法治思维深化改革、推动发展、维护稳定。强化对行政权力的制约，用制度管权管人管事，推进政府及其工作人员按照法定权限和程序行使权力，切实做到法定职责必须为、法无授权不可为，使依法办事成为政府工作人员干事创业的常态，习惯在严格遵章守纪中做好工作，习惯在严格律己中担当责任。

　　坚持依法决策。按照依法依规、集体决策、公开透明、终身负责的原则，健全公众参与、专家论证、风险评估、合法性审查、集体讨论决定等决策程序，实行政府法律顾问制度全覆盖，规范政府系统重大事项决策行为。完善重大决策合法性审查机制，规范合法性审查工作流程，未经合法性审查或经审查不合法的，不得提交集体讨论。推进重大决策风险评估制度，切实提高决策质量。自觉接受市人大及其常委会的法律监督和工作监督，主动接受市政协的民主监督，认真办理人大议案、建议和政协提案。广泛听取各民主党派、工商联和无党派人士意见，高度重视社会公众和新闻舆论监督，充分发挥工会、共青团、妇联等人民团体的重要作用。

　　规范行政执法。按照“横向集中、纵向下移”原则，推进行政执法体制改革，进一步理顺和完善工商、质监、食品药品等领域执法监管，积极探索公共卫生、安全生产、交通运输、城乡建设等领域综合执法，在公共资源交易等领域开展相对集中处罚权工作。完善行政执法程序，进一步规范执法自由裁量权。严格落实行政执法责任制，大力整治不作为、慢作为、乱作为等行为，推进严格规范、公正文明执法。重视行政复议和行政应诉，切实保障公民合法权益。

　　打造阳光政务。加大政务信息公开力度，以公开为原则、不公开为例外，推进决策、执行、管理、服务和结果公开，让权力在阳光下运行。重点推进公共资源配置、政府及部门预决算和“三公”经费预决算等信息公开，接受社会监督。建立最严格的行政审批事项准入制度，防止行政审批事项边减边增、明减暗增。整合设立市长热线服务中心，进一步畅通民意沟通渠道，及时回应社会关切。

　　推进普法守法。弘扬法治精神，深入推进“六五”普法，广泛开展“法律六进”活动，努力形成办事依法、遇事找法、解决问题用法、化解矛盾靠法的良好氛围，让法治成为全社会的共同意识和行为准则。政府系统全体人员以身作则、率先垂范，带头学法、模范守法，争做遵纪守法的表率。认真落实党风廉政建设“一岗双责”，严格执行廉洁从政各项规定，厉行勤俭节约，持之以恒反对“四风”。加强监察和审计监督，从严查处违纪违法案件，以锲而不舍、驰而不息的决心，不断提高拒腐防变和抵御风险能力，全力推进健康发展。

　　各位代表，同志们，我们已经踏上再出发、争上游的新的征程，时不我待，责在其身。让我们紧密团结在以习近平同志为总书记的党中央周围，在省委、省政府和市委的坚强领导下，进一步解放思想，改革创新，团结拼搏，开拓奋进，为开创滁州创新发展、绿色发展、健康发展的新局面而努力奋斗！
